# Biochemical analysis of catecholamine and cortisol for the evaluation of the fetal distress in third trimester stillbirths

**DOI:** 10.1007/s00414-024-03303-2

**Published:** 2024-07-30

**Authors:** Arianna Giorgetti, Maria Paola Bonasoni, Elena Lacchè, Giuseppina Comitini, Costanza Migliavacca, Alice Ferretti, Carla Galeone, Alessandra Polese, Giovanna Stridi, Francesca Monari, Beatrice Melis, Susi Pelotti

**Affiliations:** 1 Unit of Legal Medicine, Department of Medical and Surgical Sciences, University of Bologna, Via Irnerio 49, 40126 Bologna, Italy; 2 Pathology Unit, Azienda USL-IRCCS Di Reggio Emilia, Via Amendola 2, 42122 Reggio Emilia, Italy; 3 Department of Obstetrics and Gynecology, Azienda USL-IRCCS Di Reggio Emilia, Via Amendola 2, 42122 Reggio Emilia, Italy; 4 Clinical Chemistry and Endocrinology Laboratory, Department of Diagnostic Imaging and Laboratory Medicine, Azienda USL-IRCCS Di Reggio Emilia, Via Amendola 2, 42122 Reggio Emilia, Italy; 5 Obstetrics and Gynecology Unit, Mother-Infant and Adult Department of Medical and Surgical Sciences, University of Modena and Reggio Emilia, Via del Pozzo 71, 41125 Modena, Italy

**Keywords:** Forensic pathology, Intrauterine death, Prenatal stress, Epinephrine, Norepinephrine, Cortisol

## Abstract

**Background:**

Stress hormones like catecholamine and cortisol are thought to reflect the magnitude of physical stress in adults and were studied in relationship to the cause of death and agony time. Intrauterine distress, intrapartum events, and modes of delivery can affect the fetal endocrine stress response, as reflected by biochemical analyses. The aim of the present study was to evaluate the role of catecholamines and cortisol as markers of ante-mortem fetal distress. The role of cortisol as a marker of circadian timing of delivery was also assessed.

**Methods:**

A 2-year prospective cohort-comparison inclusion of stillbirths and newborns took place with collection of antemortem data, labor parameters, neonatal outcome, post-mortem data and blood samples. Stillbirths were classified as acute or chronic on the basis of a multidisciplinary evaluation. Heart blood of stillbirths and cord blood of newborns were analyzed by high pressure liquid chromatography (HPLC) for adrenaline and noradrenaline and by immunoassay for cortisol determination.

**Results:**

Fifteen stillbirths and 46 newborns, as a comparison group, delivered by spontaneous vaginal birth, elective, and emergency cesarean sections were included. Stillbirths’ main cause of death was cord thrombosis. Levels of adrenaline and noradrenaline (median: 14,188 pg/ml and 230.5 pg/ml, respectively) were significantly higher (*p* < 0.001) in stillbirths than in newborns and were also higher in acute compared to chronic distress. Cortisol levels were significantly higher (*p* < 0.05) in spontaneous vaginal delivery (median: 18.2 μg/dl) compared to elective cesarean sections (median: 3.8 μg/dl). No difference in cortisol concentrations was detected between newborns delivered at morning and at afternoon/evening.

**Conclusion:**

Our results suggest that the biochemical measurement of adrenaline and noradrenaline levels might reflect a marked physical stress response during the process of death in stillbirths. On the contrary, the elevation of cortisol levels could mirror the elevation in maternal cortisol level during vaginal delivery. For the post-mortem evaluation of stillbirths, the analysis of CA levels could provide additional data on the duration of distress, useful to integrate the forensic diagnosis.

## Introduction

Obstetrics is one of the disciplines with a greater risk for medical malpractice claims and liability proceedings, due to problems during labor and delivery involving the mother or the fetus [1, 2]. Contested definitions in this setting represent an additional challenge [3]. In fact, in a clinical context, “non-reassuring fetal status” is generally preferred over “fetal distress”, defined as a progressive fetal hypoxia and/or acidemia [4, 5]. Whatever the term used, inadequate fetal oxygenation might result in neonatal and perinatal encephalopathy, asphyxia and also fetal death [4], that is defined by the ICD-11 as the “death prior to complete expulsion or extraction from a woman” [6].

Within fetal deaths, the deaths of a baby after 28 weeks of pregnancy, before or during labor, are defined as stillbirth by the WHO [7].

A recent study in Italy has reviewed the General Register of Criminal Records of the Rome Public Prosecutor’s Office, showing that most convictions in the obstetrical field involved intrauterine fetal deaths [2]. In Belgium, France and United Kingdom, as well, a large number of legal proceedings and claims involves the timely recognition of intrapartum fetal distress and birth-related events [8–10].

On suspicion of professional obstetrical and gynecological malpractice, the identification of the exact cause and of the time of death is inextricably based on a complete post-mortem examination of the fetus and of the placenta [11]. Indeed, liability profiles could change depending on whether or not the fetus was alive at the moment of the alleged malpractice, and this might allow to understand the chances of an effective intervention and/or of survival of the fetus [3, 12].

However, an accurate estimation of the timing of onset of fetal distress and of the precise moment of death are controversial issues in forensic pathology [10, 13].

Clinical and ultrasonographic surrogate markers for asphyxia, e.g. fetal heart rate or cord blood gases, might be useful in this evaluation, though they are hampered by a rather poor predictive value and by misconception regarding the effects of intrapartum metabolic acidemia on the fetus [14].

Moreover, clinical data might lack, further limiting the ability to accurately assess the timing of death, especially with a high degree of probability or scientific certainty.

To determine when the fetus died in relation to delivery, degenerative changes occurring during intrauterine dead fetus retention, i.e. maceration timing parameters, as well as the histological evaluation of fetal organs and placenta are used [13, 15, 16], and, according to a recent systematic review, these may allow a reliable and accurate estimation [3].

The type of hypoxic-ischemic injury, whether acute, subacute or chronic, might also help in order to distinguish a prepartum vs intrapartum distress [10], since the post-mortem identification of hypoxic brain lesions suggests a certain in utero survival time [13].

In post-mortem examinations, biochemical analyses might help to assess the cause and the process of death as well as in evaluating the stress response.

Acute stress response activates the sympatho-adrenomedullary (SAM) and the hypothalamic–pituitary–adrenal (HPA) axes, stimulating a hormonal cascade that ultimately leads to the release of catecholamines (CA) and cortisol [17]. SAM and HPA axes are fundamental for maintaining homeostasis in fetuses and newborns and are stimulated by hypoxic-ischemic encephalopathy in animal models of hypoxia [18].

CA are humoral factors and neurotransmitters mainly consisting of adrenaline, noradrenaline and dopamine and, according to the literature, an increase in CA blood levels is associated with various diseases that involve neurodegeneration, traumatic brain injury, catecholamine-producing tumors and stress factors [19].

In the fetus, CA are produced by adrenal medulla as well as, to a greater extent, by the “Zuckerkandl” organ [20]. CA are thought to correlate with fetal pH and PO_2_, and increase in association with fetal distress [21, 22]. Also, CA levels might reflect different modes of delivery [20].

Cortisol is a steroid hormone secreted by the adrenal glands and typically follows a circadian rhythm, increasing during the day, with a peak around 8:30, and decreasing during the night [17, 23, 24]. Cortisol levels rise in women during pregnancy due to an up-regulation of the HPA axis, contributing to various physiological processes that support the fetal growth, development, and tissue maturation. This is also demonstrated by the fact that both insufficient and excessive levels can potentially impact fetal well-being [25], and also leading to stillbirths in animal models [26].

It has been reported that in chronic intrauterine hypoxemia, the stimulation of the HPA axis could maintain raised levels of circulating cortisol and that cortisol might represent a biomarker of prenatal stress [27].

The aim of the present study was to compare the levels of stress hormones (adrenaline, noradrenaline and cortisol) in blood of stillbirths and of newborns, to evaluate their association to the type and duration of distress and their role as markers of ante-mortem fetal distress. Furthermore, we aimed at assessing whether cortisol levels might help estimating the time of death in fetuses on the basis of its circadian release, by analyzing it in newborns delivered during the day and night.

## Materials and methods

### Study design

This observational cohort-comparison study was conducted at the Hospitals of Reggio Emilia and Modena, with a prospective inclusion of stillbirths (cohort of interest) and newborns (comparison group) delivered between January 1, 2021 to December 31, 2022. Stillbirths were included when occurred in the third trimester and submitted to a post-mortem examination. Exclusion criteria were: fetuses with congenital malformations; post-mortem interval ≥ 4 days.

As comparison, newborns delivered by spontaneous vaginal delivery (SVG), elective (ELCS), or emergency cesarean section (EMCS) were included.

Sample and data collection as well as measurements of CA and cortisol were performed for cohort and comparison groups.

The study was approved by the Ethical Committee of Area Vasta Emilia Nord (Prot. n. 2021/0074635 of 09.06.2021).

### Sample and data collection

The study involved the retrospective collection of antepartum clinical data, labor parameters, neonatal outcomes for both the cohort of interest and the comparison group, with the addition of postmortem data for stillbirths.

Antepartum clinical data collected was: maternal age; premature rupture of membranes (PROM), gestational diabetes, hypertension during gestation, intrauterine growth restriction (IUGR), prior cesarean delivery, positive swabs for Streptococcus Agalactiae, placenta previa and any other relevant information (e.g. cholestasis, obesity, pre-eclampsia etc.).

The labor parameters included gestational age at delivery, fetal heart rate abnormalities during labor or abnormal cardiotography (CTG), mode of delivery, oxytocin administration or dynamic dystocia, abnormal presentation of the fetus or mechanical dystocia.

For newborns, the neonatal outcomes collected included sex, birth weight, abnormal Apgar scores (< 8 in at least one evaluation) and time of delivery, classified as morning or afternoon/night. We considered as “morning” the period between 06:00 and 10:00 and as “afternoon/night” the period between 16:00 and 20:00 , according to the laboratory and past literature data [24]. Apgar scores and time of delivery were not available for stillbirths.

For stillbirths, a complete post-mortem examination took place, including accurate placenta and annexes pathological evaluation, and the following data was extracted: sex; birth weight; significant features at the autopsy and status of the placenta and annexes, with particular reference to chorioamnionitis and/or funisitis; cause of death, as determined after comprehensive evaluation by a pathologist with a 25-year expertise in the field of stillbirths.

After a comprehensive and multidisciplinary evaluation of all data including clinical-circumstantial data, pathological causes of death, results of histological analysis of the fetus, the placenta and the annexes, along with signs of maceration and loss of basophilia, which denote the time between intrauterine hypothetical death and delivery [15, 16], a panel of experts composed by a gynecologist (GC), a fetal pathologist (MPB) and two forensic pathologists (AG, EL) classified the type of distress, whether acute (≤ 24 h of distress) or chronic (> 24 h of distress).

Following delivery, cardiac blood from stillbirths and cord blood from newborns were sampled and submitted to biochemical analyses. All samples were processed by the laboratory within 20 min, centrifuged 10 min × 3000 g, and stored in liquid nitrogen tanks at about -196 º C until analysis.

### Biochemical analyses of catecholamines and cortisol

All biochemical analyses took place at the Hospital of Reggio Emilia. After centrifugation, serum analysis of CA, i.e. adrenaline and noradrenaline, was performed by high pressure liquid chromatography (HPLC), with a detection method (chromatographic separation and measurement by electrochemical detector) analogous to determining plasma CA in the patients’ blood at hospital.

First step was the preparation of samples, calibrator and control. A plasma volume of 1.0 ml for calibrator, control and sample was separated through selective adsorption by aluminium oxide suspension; subsequently 50 µl of Internal Standard (IS) was added into the cartridges, that were closed and shaken upside down for 10 min. Then the upper of the sample preparation column was removed, and the sample centrifuged for 1 min at 1000 g in order to discard the effluent. Subsequently, 1 ml of washing solution was put in the sample, centrifuged for 1 min at 1000 g, and the effluent was discarded. The washing procedure was carried out three times. Elution buffer (120 µl) was added to the elution vial supplied with the sample preparation column. Afterwards the eluate was mixed for 1 min on a vortex mixer and 40 µl were injected into the HPLC system.

The setting of the flow speed pump was 1 ml/min and the potential of the detector was 500 mV. The sensivity was 10 nA and the filter was set at 5 s (0.2 Hz).

The chromatograms obtained are evaluated with the internal Standard method via the peak areas/peak heights. The HPLC pump was adjusted to a flow speed of 1 ml/min, an equilibrated column for plasma catecholamines was connected to the system and the performance potential of the electrochemical detector was set at 500 mV. The range of measurements were 0.0–840 pg/ml for adrenaline and 0.0–420 pg/ml for noradrenaline.

When the values of CA were too concentrated for the instrumental analyses resulting over the range of measurements, the specimens were diluted (dilution factor) from 1:5 to 1:20 to determine the final amounts of analytes (adrenaline and noradrenaline).

Blood cortisol was determined using a commercial kit LIAISON® Analyzer, having limit of detection at 0.16 μg/dl, limit of quantification at 0.62 μg/dl, intra-assay coefficient of variations (CV%) between 5.0, 5.3 and inter-assay CV% between 4.8 and 9.3% and 99–111% of recovery.

For cortisol analysis, the standard ranges used by the analytical laboratory reflected its circadian rhythm. The morning and afternoon/night range of measurements for our laboratory were 4.5–25 µg/dl and 1.5–7 µg/dl, respectively. The levels were in accordance with the literature data [23].

For both cortisol and CA, precision was tested with serial consistent measurements, calculating the coefficient of variation which proved to be < 10%. The accuracy of the dosage was checked by 10 serial dilutions, and non-accurate data was not used.

### Data and statistical analysis

Descriptive statistics was provided for all the included data. For numerical variables, gaussian distribution was analyzed by means of the “sktest”, which takes into account skeweness and kurtosis, and identify a parametric distribution when *p* < 0.05.

When comparing cohort and comparisons, associations with categorical variables were assessed by chi-square analysis, while differences in numerical variables were evaluated by parametric or non-parametric t-test.

For biochemical analyses, CA and cortisol levels were compared between stillbirths and groups of comparison (SVG, ELCS, EMCS) by parametric or non-parametric ANOVA test and post-hoc analysis.

Within the casuistry of stillbirths, concentrations of CA and cortisol were compared on the basis of the interval between delivery and autopsy expressed in days (0 vs 1 vs 2 vs 3 days) by parametric or non-parametric ANOVA test and post-hoc analysis. Comparisons between stillbirths classified as acute and chronic took place by parametric or non-parametric t-test.

Finally, cortisol levels of newborns delivered during the day and during the afternoon/night were also compared by t-test.

For all tests, *p* < 0.05 was set for significance.

Statistics and figures were performed by Stata (StataCorp LLC, Lakeway Drive, Texas, USA, v 15.1) and Prism (GraphPad software LLC, v 9.0.0).

## Results

### Sample and data collection

Overall, 15 stillbirths and 46 newborns, delivered by SVG (*n* = 20), ELCS (*n* = 15) and EMCS (*n* = 11), were included in the study.

Results of antepartum clinical data, labor parameters, neonatal outcomes and comparisons, scattered as SVG, ELCS and EMCS, are reported in Table 1. Among stillbirths, “other antepartum clinical data” was reported in 2 cases, including one with a mother affected by congenital pituitary dwarfism and growth hormone deficiency and one case of birth of twins, one of which survived. Among controls, one case of SVG had maternal factor V Leiden thrombophilia, one fetus delivered by ELCS showed strict cord entanglement around the fetal neck and one had factor XI deficiency, while a positivity to the Coombs test was detected in one fetus delivered by EMCS.

**Table 1:** Comparisons between the cohort of stillbirths and the control group, consisting of newborns delivered by spontaneous vaginal delivery (SVG), newborns delivered by elective cesarean section (ELCS), newborns delivered by emergency cesarean section (EMCS). Maternal age, gestational age at delivery and birth weight in gram (g) are given as mean and standard deviation (SD) within brackets. CS: cesarean section; CTG: cardiotocography; IUGR: intrauterine growth restriction; PROM: premature rupture of membranes; *: statistically significant by t-test or chi-square analysis. np: analysis not performed

		*Cohort* *Stillbirths* *n* = *15*	*Comparisons*			*Chi square or t-test*
			*SVG* *n* = *20*	*ELCS* *n* = *15*	*EMCS* *n* = *11*	*p value*
*Antepartum clinical data*	Maternal age (mean + SD)	31.7 (4.9)	33.8 (4.5)	35.7 (5.6)	33.0 (5.5)	0.105
	PROM	-	6/20	-	2/11	0.083
	Gestational diabetes	1/15	1/20	2/15	1/11	0.804
	Hypertension or preeclampsia	-	3/20	1/15	2/11	0.141
	IUGR	1/15	1/20	-	-	0.077
	Prior CS	-	-	8/15	-	0.241
	Positive swabs	-	3/20	1/15	1/11	0.183
	Other	2/15	1/20	2/15	1/11	0.363
*Labor parameters*	Gestational age (mean + SD)	33.5 (5.1)	39.9 (0.7)	38.7 (1.0)	38.6 (2.8)	0.001*
	Fetal heart rate abnormalities/abnormal CTG	2/15	1/20	-	4/11	0.795
	Mode of delivery, with emergency CS	3/15	-	-	11/11	0.036*
	Oxytocin administration/dynamic dystocia	-	1/20	-	7/11	0.108
	Abnormal presentation of the fetus/ mechanical dystocia	-	-	4/15	-	0.237
*Neonatal outcomes*	Sex of the newborn	8 M; 7 F	11 M; 9F	6 M; 9F	7 M; 4F	0.711
	Birth weight in g (mean + SD)	1,877.5 (1,083.8)	3,363.2 (352.5)	3,259 (395.3)	3,262.2 (585.8)	0.001*
	Abnormal Apgar scores (< 8)	-	1/20	0/15	3/11	np

The cohort of interest and the comparisons did not show a statistically significant difference and were not statistically associated with any of the antepartum clinical data.

Considering labor parameters, gestational age was statistically lower in stillbirths compared to the control groups, while emergency cesarean section was significantly more frequent in the group of the comparisons, and particularly in the EMCS group.

Among neonatal outcomes, males and females were equally represented in the cohort of interest and in the comparison groups. Weight at birth was significantly lower in stillbirths compared to controls and abnormal Apgar scores were not compared. Among newborns, 22 were delivered during the morning, and 20 during the afternoon/night period, considering our classification.

Causes of death in stillbirths were as follows: abnormalities involving the cord and cord thrombosis in 8/15 (53.3%), abruptio placentae in 3/15 (20%), placental insufficiency in 3/15 (20%, including 2 cases of placental infarct, 1 of villous dysmaturity) and 1/15 case (6.7%) showing both placental insufficiency and cord thrombosis.

After multidisciplinary classification on the timing of distress, whether ≤ 24 h, or > 24 h, stillbirths were classified as acute in 9/15 cases (60%) and chronic in 6/15 (40%) (Table 1).

**Fig. 1:**
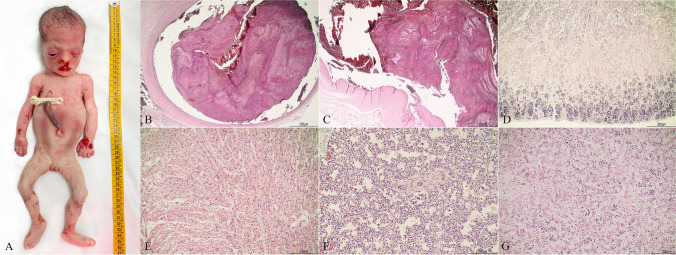
Example of acute type of distress (case #12). **A** Fetal external examination showing minimal maceration with focal skin slippage in the head, upper, and lower limbs. **B** Chorionic vessel thrombosis: non-occlusive initially laminated thrombi were found in many vessels (2x). **C** Umbilical cord thrombosis: a non-occlusive thrombus with lamination was observed at cord restriction (2x). **D** Kidney histology: loss of nuclear basophilia was mainly present in the medulla with cortical preservation in tubules and nephrogenic line (4x). **E** Heart histology: myocardiocytes presenting well preserved nuclear basophilia (10x). **F**. Lung histology: nuclear basophilia was overall maintained within bronchi and alveoli (10x). **G** liver histology: hepatocytes with well identifiable nuclear basophilia (10x)

**Fig. 2:**
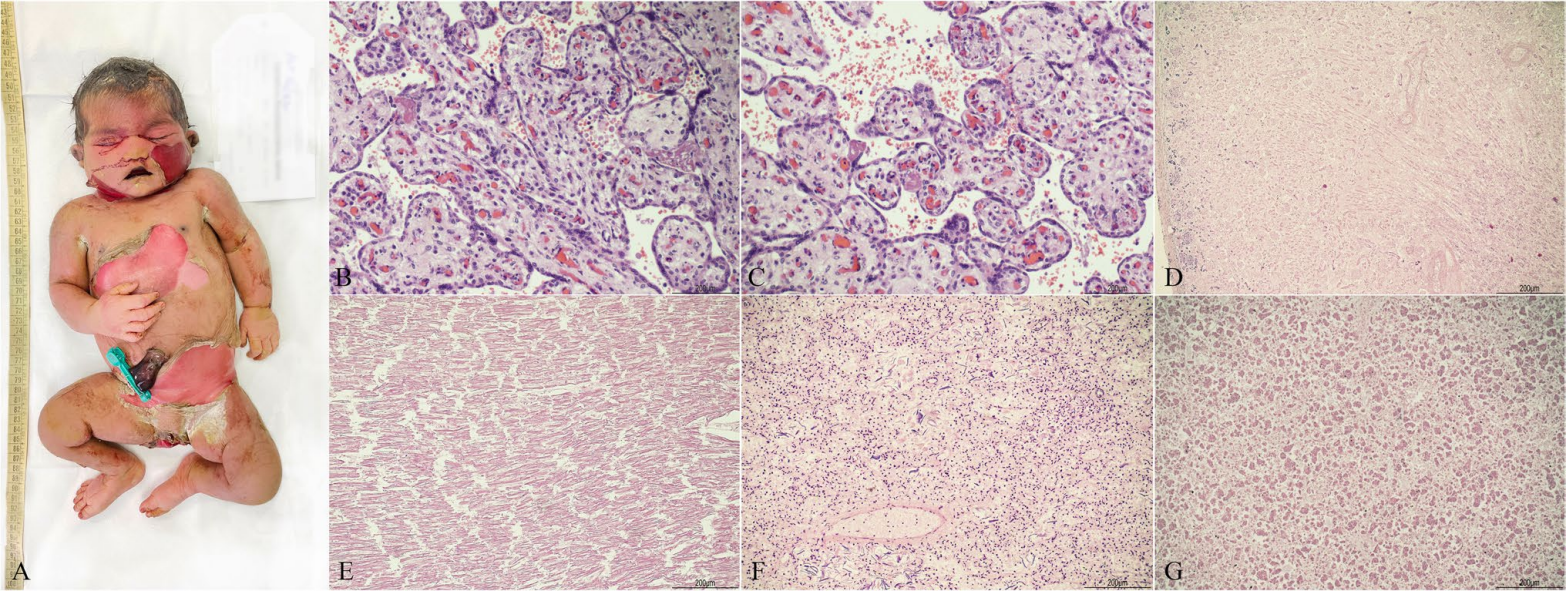
Example of a chronic type of distress (case #2). **A** Fetal macroscopical examination showing intermediate maceration with wide skin slippage in the face, torso, abdomen, upper, and lower limbs. **B** and **C** Placental histological analysis exhibited typical features of maternal gestational diabetes with diffuse villous dysmaturity characterized by enlarged villi with irregular contour and highly reduced vasculo-syncytial membranes (20x). **D** Kidney histology: nuclear basophilia was weakly maintained in the outer part of the nephrogenic line only (4x). **E** Heart histology: myocardiocytes were fragmented with absence of nuclear basophilia (10x). **F** Lungs histology: respiratory airways presented widespread disepithelization and scarce preservation of nuclear basophilia (10x). **G** liver histology: hepatocytes showed a loose structure with complete loss of nuclear basophilia (20x)

More detailed results on post-mortem data and multidisciplinary classification of stillbirths in acute or chronic are reported in Table 2. Figures 1 and 2 represent examples of the two types of distress, among the here-presented casuistry.

**Table 2:** Case series of stillbirths, showing the cause of death, the macroscopical and microscopical findings of the post-mortem examination of fetus, placenta and annexes, and the classification of the type of distress

*Case n*	*Cause of death*	*Additional macroscopical findings including placenta and annexes examination*	*Additional histological findings*	*Type of distress*
*#1*	Placental insufficiency due to multiple placental infarcts	One true knot of the cord	-	Chronic
*#2*	Placental insufficiency due to diffuse villous dysmaturity	Intermediate maceration, meconium staining	Chorioamnionitis stage 2/3, grade 1/2	Chronic
*#3*	Cord thrombosis in hypercoiling and abnormal insertion (eccentric, oblique with amniotic band)	Minimal maceration, meconium staining, 8 coils/10 cm	Chorioamnionitis stage 1/3, grade 1/2	Acute
*#4*	Placental insufficiency due to diffuse infarcts (90%)	Advanced maceration	-	Chronic
*#5*	Umbilical vein thrombosis and cord entanglement around fetal parts	-	-	Acute
*#6*	Abruptio placentae	-	-	Acute
*#7*	Cord thrombosis at insertion in furcate cord	-	-	Acute
*#8*	Umbilical vein and chorionic vessel thrombosis; furcate and oblique cord, with amniotic band; placental insufficiency with multiple placental infarcts, involving 50% of the placental volume	Advanced maceration	Chorioamnionitis stage 2/3, grade 1/2; multiple recent, intermediate placental infarcts	Chronic
*#9*	Umbilical vein thrombosis at the cord insertion in hypercoiling	Advanced maceration, 7 coils/10 cm	Subchorionitis	Chronic
*#10*	Umbilical vein and artery thrombosis at cord insertion and abnormal insertion (oblique with amniotic band)	Meconium staining, minimal maceration	Subchorionitis	Acute
*#11*	Umbilical vein and artery thrombosis at cord insertion and abnormal insertion (oblique with amniotic band)	Meconium staining, minimal maceration	Subchorionitis	Acute
*#12*	Cord thrombosis in fetal restricted fetal end and furcate cord	Pleural and pericardial petechiae, minimal maceration	Chorioamnionitis stage 2/3, grade 1/2; few chorionic vessels with thrombosis	Acute
*#13*	Placental abruptio	-	-	Acute
*#14*	Cord restrictions in hypercoiling	Advanced maceration	-	Chronic
*#15*	Placental abruptio	Minimal maceration	-	Acute

### Biochemical analyses of catecholamines and cortisol

Cardiac blood collected from 15 stillbirths and cord blood from 46 newborns were submitted to biochemical analyses, for a total of 61 samples.

Adrenaline showed a normal distribution (*p* < 0.05) at sktest, while noradrenaline and cortisol did not, therefore non-parametric statistics were used for all analyses.

Median adrenaline levels in stillbirths and in comparisons, taken all together, were 14,188 pg/ml (IQ = 3,577.6–48,350) and 230.5 pg/ml (IQ 67.4–465.0). Adrenaline levels of stillbirths were significantly higher (*p* < 0.0001) than comparisons, even when singularly considering SVG, ELCS, EMCS.

Noradrenaline median levels were 70,140 pg/ml (IQ = 46,740–137,873) in stillbirths and 484.3 pg/ml (IQ = 359.8–719.0) in the overall comparison group, showing a statistically significant difference (*p* < 0.001). The statistically significant difference was confirmed when considering each comparison group (SVG, ELCS, EMCS) separately.

The levels of adrenaline and noradrenaline between each group of comparison and the others, on the contrary, were deemed non-statistically significant.

**Fig. 3:**
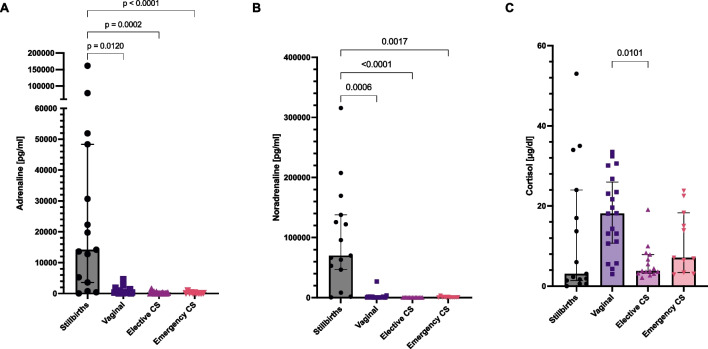
Results of the analysis of adrenaline (**A**), noradrenaline (**B**) and cortisol (**C**) in the cohort of interest (stillbirths) and in the comparison group: vaginal delivery, elective cesarean section (CS) and emergency CS. Significant comparisons are shown with their respective p values

Cortisol cord levels did not show a statistically significant difference between the cohort of interest of stillbirths (median 3.1 μg/dl, IQ = 1.4–24.0) and comparisons taken all together (*p* > 0.05). Within the comparison groups, cortisol was significantly higher (*p* < 0.05) in spontaneous vaginal delivery (median: 18.2 μg/dl, IQ = 10.7–26.0) compared to elective cesarean sections (median: 3.8 μg/dl, IQ = 3.2–7.9). Details are given in Fig. 3.

Adrenaline, noradrenaline and cortisol showed no statistically significant difference in stillbirths when compared on the basis of the interval between delivery and autopsy expressed, and results are shown in Table 3.

**Table 3:** Results are given as median and interquartile range between brackets

	Interval in days between delivery and autopsy				*p value*
	0 day	1 day	2 days	3 days	
*Adrenaline* *[pg/ml]*	3,578 (424–48,350)	19,750 (9,721–106,679)	6,772 (222.2–13,423)	30,691 (22,320–78,650)	0.0782
*Noradrenaline* *[pg/ml]*	53,009 (1,665–315,668)	137,873 (65,263–188,585)	66,730 (16,250–89,490)	66,558 (46,740–125,750)	0.5328
*Cortisol* *[μg/dl]*	6.0 (3.1–13.7)	1.9 (1.2–18.2)	26.0 (5.3–48.5)	0.6 (0.0–24.0)	0.3335

Median adrenaline in acute and chronic stillbirths were 30,691 pg/ml (IQ = 13,205–65,273) and 4,416 pg/ml (IQ 327.6–16,221). For noradrenaline, median concentrations were 122,150 pg/ml (IQ = 68,349–188,585) and 27,558 pg/ml (IQ 1,384–74,225)

**Fig. 4:**
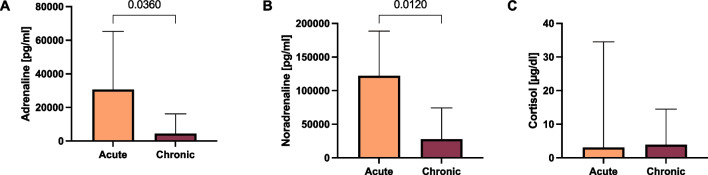
Comparison of adrenaline (**A**), noradrenaline (**B**) and cortisol (**C**) levels between stillbirths classified as acute and chronic. Significant comparisons are shown with their respective p values

Catecholamines were significantly higher in acute stillbirths compared to chronic deaths with *p* < 0.05 (Fig. 4)

Median adrenaline in acute and chronic stillbirths were 30,691 pg/ml (IQ = 13,205–65,273) and 4,416 pg/ml (IQ 327.6–16,221). For noradrenaline, median concentrations were 122,150 pg/ml (IQ = 68,349–188,585) and 27,558 pg/ml (IQ 1,384–74,225).

Catecholamines were significantly higher in acute stillbirths compared to chronic deaths with *p* < 0.05 (Fig. 4).

For cortisol, on the contrary, levels between acute, median 3.1 μg/dl (IQ = 1–34.5), and chronic stillbirths, 3.95 μg/dl (IQ = 1.4–14.5), did not show a significant difference (*p* > 0.05).

**Fig. 5:**
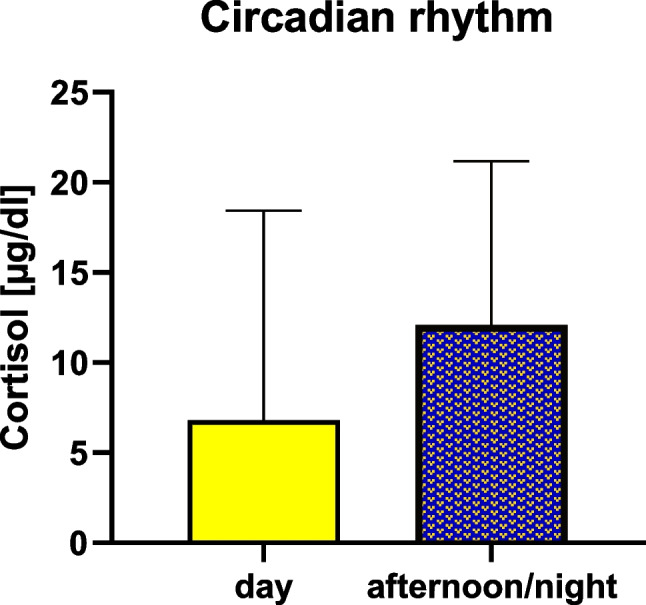
Comparison between newborns delivered during the day and during the afternoon/night

When considering the circadian rhythm of cortisol within the comparison group, newborns delivered during the morning (*n* = 22) had a median amount of 6.8 μg/dl (IQ = 3.6–18.4) and those delivered during the afternoon/night (*n* = 20) showed cortisol median level of 12.1 μg/dl (IQ = 4.2–21.2). No statistically significant difference (*p* > 0.05) was found between those concentrations (Fig. 5).

The difference tested non-significant also when considering separately the groups of SVG, ELCS, EMCS (*p* > 0.05). The comparison was not performed for stillbirths, given the uncertain time of death.

## Discussion

Here we report a casuistry of 15 stillbirths and 46 newborns, used as cohort of interest and comparisons, respectively. As expected, stillbirths and newborn did not significantly differ except for the gestational age and weight at birth, since the included stillbirths all occurred in the third trimester, while the included newborns were closer or at the physiological term of the pregnancy.

The absence of significant associations between the cohort/comparison and antepartum clinical data, labor parameters or neonatal outcomes suggests that there are no spurious factors that could affect the biochemical analyses, except for those shown in Table 1, i.e. gestational age, mode of delivery with emergency CS and birth weight.

### Catecholamines

Serum and urinary CA have been studied in adult humans as biomarkers of cause of death and agonal time for forensic purposes, showing sometimes contradictory results [28–30]. Indeed, the interpretation of biochemical analyses in the post-mortem setting could be affected by interference due to pre-existing factors disorders during life, cause of death, survival period, post-mortem changes (e.g., autolysis and protein degradation), environmental factors, chemical properties, distributions, and locations of the analytes and analytical procedures [31, 32]. The major drawback in the dosage of CA has been reported as the poor stability at room temperature and the degradation connected to post-mortem changes [32, 33].

In the present study, CA in blood of stillbirths were significantly higher than in blood of newborns and exceeded clinical reference intervals, requiring dilutions. This seems to indicate a stability or a significant release of adrenaline and noradrenaline in the peri-mortem period, rather than a degradation. A stability of CA up to 60 h of storage in a cool environment was shown by Berg and Bonte [34]. In another study, adrenaline showed a mild tendency toward post-mortem increase, but this was not observed for cardiac blood and was not confirmed by further research [29, 35]. A study on 11 human cadavers detected a significant post-mortem increase of noradrenaline in heart blood from the first to the third day postmortem [35]. However, in our casuistry with comparable sample size, this was not confirmed when testing levels of CA with different interval between delivery and autopsy (Table 3).

Our results are consistent with the findings of Zhu et al. [29], who detected no significant changes in CA levels in cadaveric blood samples at ambient temperature and showed reproducible measurements for frozen samples. The findings are also consistent with those of Lee et al. [36], who highlighted no blood CA differences with various post-mortem intervals. Moreover, they seem to confirm the trials performed on cadaveric and healthy volunteers’ blood at room and refrigerated temperature, that demonstrated a reliable CA determination up to 72 h, despite a moderate decline [30].

If an increase in the delivery-autopsy interval is not explanatory of the findings, then the higher levels of CA detected in stillbirths in our casuistry might be connected to the processes leading to death, in the absence of other possible explanatory factors at the post-mortem examination.

Stillbirths in the third trimester mostly involve acidosis, systemic hypoxia, and ultimately an asphyxia mechanism. Some studies on adult humans did not find a distinct CA profile in relationship with the cause of death [30, 37], while others have shown a significantly higher amount of adrenaline and noradrenaline in deaths due to injuries, hyperthermia, poisonings and asphyxiation [29]. Lee et al. found higher femoral noradrenaline in asphyxia compared to deaths due to injuries [36]. In fatal asphyxia, systemic hypoxia and acidosis were considered major triggers for the release of CA, occurring from sympathetic nerve terminals in the extremities. The peripheral release explained why, in asphyxiation, CA blood levels from the external iliac veins were higher than those from the left heart [29].

Our results, although performed on stillbirths and not on adults, might suggest that a post-mortem increase of CA is indicative of the stress reaction during the processes leading the fetus to intrauterine death.

Within stillbirths, CA levels were significantly higher in acute death compared to cases with advanced fetal maceration and placental insufficiency, classified as chronic stillbirths. This might reflect a difference related to the agony time.

In adult autopsies, CA concentrations were shown to be elevated in prolonged agony time, with levels comparable to humans under acute maximal stress [34, 38]. Wilke et al. on the contrary found no significant difference of CA heart blood levels in relation to the length of agony, although mean noradrenaline values were higher in deceased with a short agony compares to the group with long agony [30]. Studies on strangulated animals showed significant higher levels of noradrenaline and adrenaline compared to the control (intoxicated) group (noradrenaline 5.4 ± 2.6 ng/mL vs. 2.8 ± 0.1 ng/ml; adrenaline 6.0 ± 3.4 ng/ml vs. 3.8 ± 3.0 ng/mL), suggesting a possible application of the detection of postmortem catecholamines serum levels in establishing the agonal period. In the same study, CA were suggested as good markers of hypoxia or acute stress, even without pain [28].

Despite the limited number of observed stillbirths, our results seem to be in line with these animal models, suggesting that CA could represent a promising biomarker for acute fetal distress. In chronic stillbirths, CA might be released to a lower extent and/or have more time to be degraded in uterus before analysis.

### Cortisol

Although glucocorticoids are lipophilic and thus are believed to freely cross the placenta from the maternal to fetal circulation, cortisol levels are controlled in fetuses by the fetal HPA axis and by the placental enzyme 11-β-hydroxysteroid dehydrogenase-type 2 (11β-HSD2), which catalyzes the conversion of active cortisol into inactive cortisone [25, 39, 40]. There is scientific evidence that the activity of the enzyme, whose expression increases at term, is reduced by several pathological conditions, e.g., pre-eclampsia or IUGR, as well as by maternal prenatal stress, permitting a greater amount of maternal cortisol to freely pass the placenta [25, 41, 42]. However, in a recent large study, maternal stress induced a reduction, not an increase, in cortisol cord levels, likely due to blunted fetal response [43]. Placenta also releases corticotropin-releasing hormone (CRH), that can stimulate either the maternal or fetal pituitary adrenal axis, further complicating the fetal regulation of cortisol [44].

In our study, comparing cortisol levels between the stillbirths and comparisons, we did not observe a statistically significant difference. This might be due to the significantly lower gestational age of stillbirths compared to newborns, which might result in an immature HPA axis, unable to increase the production of cortisol despite illnesses [27]. Accordingly, fetal animal studies have shown that in preterm fetuses the cortisol levels, in response to moderate hypoxia or short-lasting asphyxia, are lower than in late-gestation fetuses [27].

However, severe asphyxia caused by 25-min long umbilical cord occlusion caused an upsurge of cortisol even in preterm fetal sheep, but cortisol rapidly returned to basal levels within 72 h [45]. A sustained cortisol elevation is found in ovine fetuses exposed to chronic intrauterine hypoxemia [46].

In our study cortisol levels were not statistically higher in chronic compared to acute stillbirths, suggesting that cortisol is not a good post-mortem marker of fetal distress or acute vs chronic agony. This result is rather in line with previous studies on human infants, showing that CRH, but not cortisol, was increased in cord plasma in cases of chronic fetal distress [44]. This might be due to the complex mechanisms of cortisol balancing involving the placenta and to the presence of variable contributions from fetuses and mothers [25].

A post-mortem decrease of cortisol due to instability is not confirmed by our data, but cannot be totally excluded, given its demonstrated decrease after 48 h at 4 °C [47].

Within our comparison group, cortisol was significantly higher in SVG compared to ELCS, which is consistent with past studies on preterm [44] and term infants [48], suggesting that the stress at delivery might have an influence on the infant HPA axis. Accordingly, the study of Miller et al. on 172 primiparous women with uncomplicated singleton pregnancies found higher cortisol in newborns delivered vaginally than those delivered by caesarean section [49].

The consistency of the results in newborns with past literature data further confirms the reliability of our data.

In the literature, it is known that cortisol has a circadian rhythm, showing normal levels during the early morning hours (8–10 a.m.) and decreasing in the evening and during the early sleep phase (nadir at approximately 4 a.m.), further complicating the use of the hormone as a biomarker for chronic stress [24, 50]. If this rhythm could be confirmed in fetuses, cortisol measurements could provide a clue regarding the timing of death of stillbirths.

However, since the true timing of death in stillbirths is unknown, in our study we evaluated whether cortisol blood levels in newborns could reflect the time of birth, whether delivered during the morning or the afternoon/night.

Only a few studies in the literature evaluated the cortisol circadian rhythm with respect to the time of delivery. Su et al. found low levels of cortisol in samples collected during both daytime and nighttime, although all the cases displayed chronic stress with related adrenal hyporesponsivity, which could have biased the casuistry [43].

In our study, although more research on a wider casuistry is needed, the absence of a significant difference in cortisol levels between newborns delivered at daytime and nighttime does not even allow the application of cortisol analysis to stillbirths for the evaluation of the time of death.

## Limitations

Our study presents several limitations, firstly regarding the comparison between stillbirths and newborns, performed by analyzing cardiac and cord blood, respectively. We acknowledge that the latter might more closely resemble peripheral blood and that the site of collection might have an influence on CA levels. However, the collection of peripheral blood was not possible in cases of stillbirths, due to the limited availability of this biological material during post-mortem examination of fetuses. Nevertheless, previous studies on topographical distribution demonstrated even higher CA levels in peripheral blood compared to heart blood of asphyxiated humans [29], so that even higher levels of CA could be hypothesized if cord blood was collected for stillbirths. Additionally, femoral and heart blood CA showed close relationship in a recent study [36].

The same analytical method was also used for post-mortem and ante-mortem blood, although samples were processed as soon as possible, and this could have also partially biased the results.

A third major drawback of the study resides in the classification of the type of fetal distress, due to the absence of consensus or guidelines and the difficulties in the exact determination of the time of death. In our study, we classified cases on the basis of a multidisciplinary and comprehensive evaluation of clinical-circumstantial, post-mortem data, including cause of death and histology, which is at the moment the only means to achieve a definition of acute or chronic distress. Nevertheless, we acknowledge that an acute death followed by prolonged in utero retention cannot be excluded with certainty.

A further limitation resides in the absence of maternal sampling, therefore a comparison between maternal and fetal or newborn cortisol levels was not carried out, although the impact of maternal cortisol levels was not the aim of our study and our results do not seem to suggest any need for further deepening.

Lastly, further research on a wider casuistry is strongly encouraged.

## Conclusions

In this study we presented a casuistry of third trimester intrauterine deaths, used as cohort of interest, compared to newborns delivered by vaginal or cesarean section. Our results suggest that the biochemical post-mortem measurement of adrenaline and noradrenaline might reflect a marked physical stress response during the process of death in stillbirths and that CA could be suggested as a biomarker of acute fetal stress. Cortisol does not appear as a promising biomarker for acute or chronic fetal distress and it was not confirmed as significantly affected by the circadian rhythm at delivery. For the post-mortem evaluation of stillbirths, the analysis of CA levels could provide additional data on the duration of distress, useful to integrate the forensic diagnosis.

## Data Availability

The datasets generated during and/or analysed during the current study are available from the corresponding author on reasonable request.
